# Establishing a reasonable price for an orphan drug

**DOI:** 10.1186/s12962-020-00223-x

**Published:** 2020-09-04

**Authors:** Mikel Berdud, Michael Drummond, Adrian Towse

**Affiliations:** 1 Office of Health Economics, Southside 7th, 105 Victoria St., London, SW1E 6QT UK; 2 Centre for Health Economics, University of York, Alcuin A Block, Heslington, York, YO10 5DD UK

**Keywords:** Orphan drugs, Reasonable price, Value-based pricing, Rate of return, Cost-effectiveness threshold, R&D costs

## Abstract

**Background:**

This paper addresses the question of what a reasonable price for an orphan drug is. The research proposes a way to adjust an established payer/HTA body incremental cost-effectiveness threshold (CET) to take account of differences in patient populations and costs of research and development in order to sustain prices that generate rates of return from investments in developing orphan drugs that are no greater than the industry average.

**Methods:**

We investigated the cost of conducting research for orphan drugs as compared to non-orphan drugs, as well as patient population sizes targeted by orphans and non-orphans. We provided an empirical illustration based on novel drug approvals of orphan and non-orphan drugs of the FDA between 2011 and 2015 (N = 182).

**Results:**

Using, for illustration, the NICE incremental CET (£20 K per QALY) as an anchor and adjusting by R&D costs and expected market revenue, we estimated the adjusted reasonable CET for orphan drugs to be £39.1 K per QALY at the orphan population cut-off and £78.3 K per QALY at the orphan population mid-point. For ultra-orphan drugs the adjusted CET was £937.1 K.

**Conclusions:**

We propose one general method for establishing a reasonable price for an orphan drug, based on the proposition that rates of return for investments in developing orphan drugs should not be greater than the industry average. More research is required on data and assumptions, but with the data and assumptions we use, we find that in order to secure such a reasonable price for an orphan drug, the CET for orphans would need to be higher. This could be one approach for establishing the maximum allowable price society should be willing to pay, although decision-makers may still wish to negotiate a lower price, or refuse to pay such a premium over the value-based price in order to treat these groups of patients.

## Background

The high cost of drugs for rare diseases (often known as orphan drugs) has generated considerable debate.^1^Many health economists argue that there is no justification for a premium for ‘rarity’ and that, in terms of reimbursement decisions (i.e. public subsidy), orphan drugs should not be judged any differently from drugs for common diseases. Given the current trend towards value-based pricing, this implies that orphan drugs should demonstrate that they represent good value for money when judged by conventional criteria. Otherwise, society would be sacrificing overall health gain in order to make these therapies available [1]. However, in practice this policy would lead to most orphan drugs being denied reimbursement [2].

Other economists have argued that there may be characteristics of orphan drugs that might justify departing from the standard value for money criteria [3]. These additional characteristics could relate to the severity of the health condition and the absence of alternative effective therapies [4]. However, surveys of the general public mostly suggest that there is no willingness to pay a premium for rarity, although there may be a case for paying more for drugs to treat severe conditions, or where there is unmet need [5, 6].

Although the question of whether society should allow the reimbursement of orphan drugs is an important issue, the reality is that orphan drugs are currently being reimbursed in many jurisdictions [7]. In some jurisdictions they are not subjected to the same level of scrutiny, through health technology assessment (HTA), as other drugs. In jurisdictions that apply HTA broadly to most health technologies, the willingness-to-pay cost-effectiveness threshold (CET) may be set at a higher level [8]. However, Coté and Keating [9] argue that there is a risk that manufacturers will exploit society’s willingness to pay for therapy in situations where individuals have no other effective therapy. They argue that many orphan drugs appear to be very profitable to manufacturers, that manufacturers may deliberately create situations whereby their drug could be designated ‘orphan’ and that many orphan drugs are marketed for multiple indications, which taken in their totality would not lead the drug to be designated orphan. In support of these arguments, Hughes and Polleti-Hughes [10] estimate that companies holding orphan drug market authorizations generate a higher return on assets.

If health care decision-makers are to provide funding for orphan drugs, they require reassurance that the prices being charged by manufacturers are not ‘excessive’. Different jurisdictions approach the pricing of drugs in different ways. In France and Germany, the price is determined by an assessment of the clinical data, followed by a negotiation. In several other European countries, including the United Kingdom, a ‘valued-based price’ is determined by assessing the cost-effectiveness of the drug and comparing this with the decision-maker’s CET, which represents the maximum amount that decision-makers would be willing to pay for given unit of health gain (such as a quality-adjusted life-year). Of course, the value-based pricing rule could be supplemented by negotiation, or, as indicated above, the CET could be set at a higher level for orphan drugs. However, in making these adjustments decision-makers would still need some ‘benchmarks’ to use in a negotiation.

One possible benchmark might be one based on the proposition that the manufacturers of orphan drugs should not make higher profits than manufacturers of drugs for non-orphan conditions. That is, the prices paid should not lead to rates of return from investments in developing orphan drugs in excess of the pharmaceutical industry average, after adjustments for risk and any other relevant factors. Using the UK as an example, this paper illustrates the implied adjustment that would need to be made to the CET should decision-makers wish to use this as a guide for setting prices.

## Methods

The two major differences between orphan and non-orphan drugs are that (i) the costs of research and development are likely to be lower for orphan drugs, as the clinical development programme is less extensive,^2^ and (ii) the treatment populations for orphan drugs are likely to be smaller, given the rarity of disease. Therefore, in order to determine a reasonable price for an orphan drug, we investigated the cost of conducting research into rare diseases, as compared with non-orphan conditions. We then investigated the adjustment that would need to be made to a payer’s “normal” CET for non-orphan drugs in order to achieve the industry-wide rate of return, in relation to the expected size of the treatment population. For illustrative purposes we use the UK threshold used by National Institute for Health and Care Excellence (NICE) in England and Wales and the Scottish Medicines Consortium (SMC) in Scotland and relative population numbers for the orphan and non-orphan treatments reviewed by these two bodies.

### A reasonable price for an orphan drug

We based our proposed approach on the assumption that, as the target population size of a medicine goes down, the revenue generated also goes down unless the drug price increases to counter the effect of lower sales volumes. On the other hand, it is also likely that the R&D cost of a drug for a rare disease, is lower than for a non-orphan drug, because smaller numbers of patients are available for recruitment to clinical trials.

We propose a reasonable price as one generated by these two opposing effects, affecting, on the one hand, revenue, and, on the other hand, the cost of drug development and commercialisation, in a way that its rate of return is approximately equal to the rate of return of a drug for a common disease.

Rather than just estimating how much higher or lower the price of an orphan drug should be because of these opposing adjustments, we have expressed the reasonable price in terms of the change that might be required in the CET which determines the maximum allowable price for a drug. The reason for this approach is that one would expect all drugs to produce health-related gains, whether designated orphan or not, but the ‘acceptable’ level of that threshold may vary depending on the designation. For simplicity we assume that all of the benefits of drugs can be expressed in QALYs. A formal development of our approach is available in Appendix 1 of this paper. In the appendix a general equation is presented in the first instance in Eq. (4) and then a simpler approach is derived from it in Eqs. (5) and (6) by applying asssumptions. The simpler approach is used for the practical adjustment exercise presented in the following sections of the paper.

Finally, it should be noted that the general approach—even in its simple form—considers global figures of both costs and revenues for non-orphan and orphan developers. In applying the approach to a particular country or market, these estimates will need to be appropriately adjusted to reflect the country’s share of the global market. We make additional assumptions to implement the general approach to a particular country or market. We assume that (i) the CET in any given country has been appropriately determined^3^ (ii) the ratios of patient numbers for a typical orphan or ultra-orphan drug, as compared to a non-orphan in a particular country are the relevant ratios and (iii) the share of the total global pharmaceutical market for all products represented by a country’s market is the relevant share of global R&D that should be charged to that market.^4^^,^^5^

### Research and development cost

All novel drug approvals completed by the Food and Drug Administration (FDA) in the period 2011–2015 were considered for the study (n = 182).^6^ The sample was divided into two groups: those approved under the orphan designation, and those approved under non-orphan designations. Within the orphan and non-orphan drug groups, the sample was also divided into oncology and non-oncology drugs, since in many jurisdictions oncology drugs indicated for small patient populations (possibly because of targeted therapy), are not treated differently for reimbursement, even though they may be technically ‘orphan’.

In order to estimate any difference between the cost of developing an orphan and a non-orphan drug, we investigated the cost of conducting research for all novel drug approvals issued in 2015. For this subsample, data on the number of patients involved in clinical trials was collected from *ClinicalTrials.gov.*^7^ For each drug, we identified the number of patients in clinical trials involved at the different phases of development (e.g. phases I, II and III) for the molecule designated as orphan drug and the specific orphan indication. Within medicines designated as orphan and non-orphan, we also distinguished between medicines approved for oncology and non-oncology indications.

**Fig. 1:**
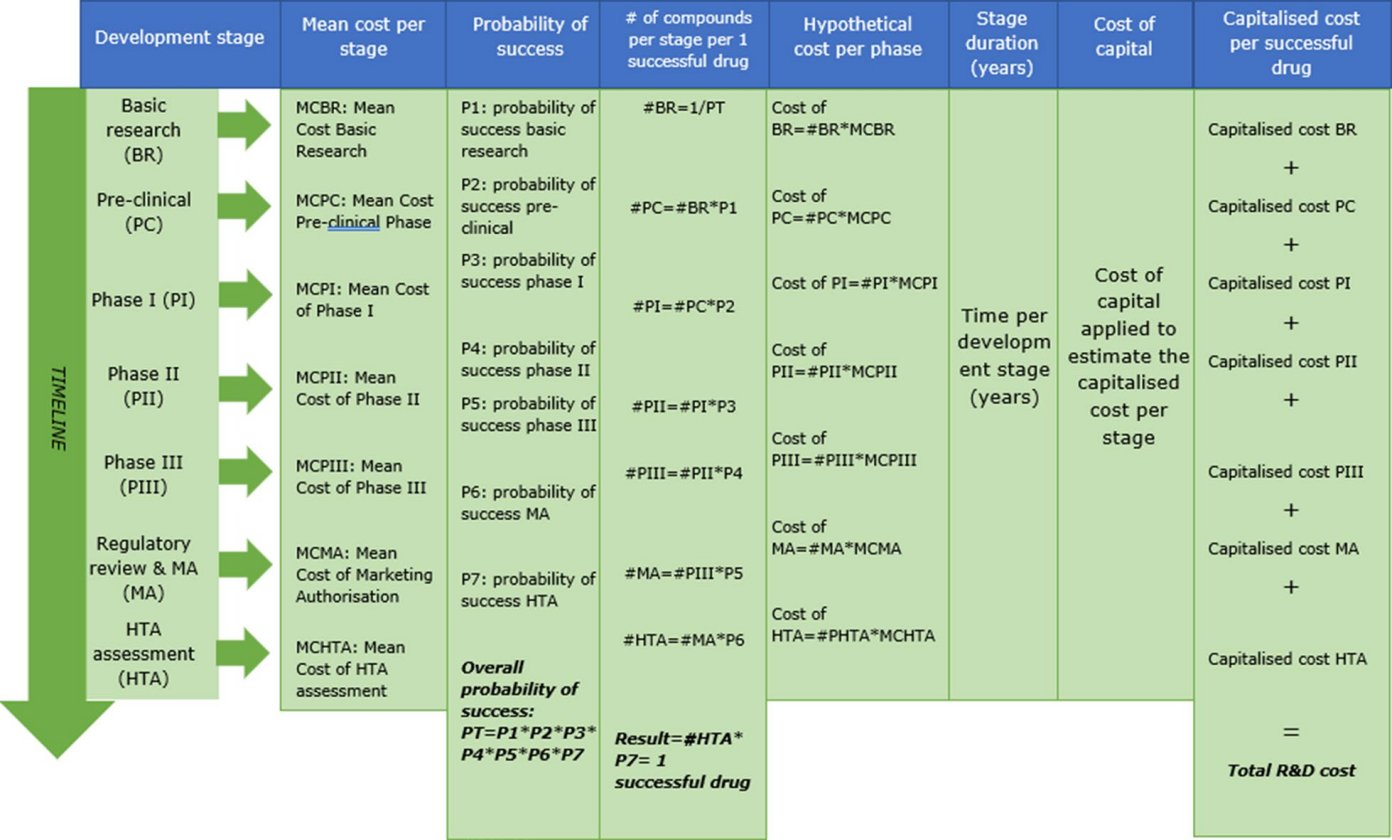
Summary of Mestre-Ferrandiz et al. [14] method to estimate the R&D cost of a new medicine (Source: Authors research based on Mestre-Ferrandiz et al. [14])

For the estimation of the cost of developing a drug we used the estimates produced by Mestre-Ferrandiz and colleagues [14] who followed the standard methodology established in related literature [15, 16].^8^ Mestre-Ferrandiz and colleagues estimate the R&D cost of a new drug based on the impact of four cost drivers: out-of-pocket costs, time of development, cost of capital, and failure rates of development. Figure 1 shows a detailed explanation of the estimation method.

We searched the literature for estimates of the per-patient cost in clinical trials. We found a per-patient trial cost—only trial site related costs—in the report by Batelle Memorial Institute [18] for Pharmaceutical Research and Manufacturers of America (PhRMA).^9^ We estimated the out-of-pocket cost by multiplying average number of patients in clinical trials and per-patient costs. We extracted data of Probability of Success (PoS) from the Biomedtracker, Pharmapremia database for the period 9/2009–9/2019. This is the same database and methodology used for other published studies [20, 21] updated to cover the most recent 10 year period.^10^ PoS for orphan drugs are estimated based on a sample of 1206 clinical trials (525 for oncology orphans). PoS for non-orphans is estimated based on a sample of 7746 clinical trials (2473 for oncology non-orphans). Finally, we kept unchanged the cost-of-capital from the original modelling of Mestre-Ferrandiz and colleagues [14] because this input has not been subject to significant changes in newer estimations of the cost of R&D using similar methods and published in the literature [16, 17]. We updated the model with the most recent estimates of the time of development for development phases 1, 2 and 3 [16]. As times for clinical development for orphan drugs and non-orphan drugs are not considered to be significantly different [23], we have used the same estimates for both.

Although very important, the costs of R&D are only one component of the cost of bringing a new drug to market. In addition, there are costs in manufacturing, marketing and distribution. It is not known whether these other costs are also lower for orphan drugs, or whether they are the same as for non-orphan products. Therefore, different assumptions were made about the potential reduction in these other costs for orphans and ultra-orphans and their impact explored in a sensitivity analysis.

### Patient population size

In order to adjust the CET (or the price), by the volumes for companies developing orphan drugs, we searched for data on target patient populations of both, orphan and non-orphan drugs. To gather this information, we consulted SMC and NICE appraisals, which often give estimates of the potential treatment population for the technologies being appraised.^11^ We considered all technology appraisals conducted during the period January 2011–March 2017.

At the time of the study, some drugs for rare conditions were being appraised by the Advisory Group for National Specialised Services (AGNSS), (see https://www.nice.org.uk/news/article/nice-to-assess-high-cost-drugs-for-rare-conditions). NICE was, however, appraising some cancer drugs for small patient populations that were designated ‘orphan’. We found data for patient populations from a total number of 48 SMC appraisals (24 orphans and 24 non-orphans) and 33 NICE appraisals (11 orphans and 22 non-orphans). Drugs appraised by both SMC and NICE, amounted to a total number of 21 drugs (7 orphans and 14 non-orphans).

In order to make the patient population data from both sources comparable we standardised by dividing by the total population of England^12^ and Scotland,^13^ and then multiplying by 50,000 to make the resulting rates per 50,000 comparable with the European Union orphan designation. Finally, although we assumed that orphan drugs would achieve sales to 100% of the potential patient population, for non-orphans we assumed that they would only achieve sales to 50%, because of the potential for in-class competition.

### Cost-effectiveness estimates and appraisal decisions

We also obtained data on the incremental cost effectiveness ratios (ICERs) of appraised medicines included in our sample, along with the appraisal decisions (recommended or not recommended). The main purpose of collecting actual ICERs and decisions was to understand what NICE and SMC had actually decided and discuss these decisions in relation to our adjusted CET method to establish a proposed reasonable price for an orphan drug.

Health technology appraisals of NICE often present more than one ICER. In such cases we followed the algorithm developed in [24] for the selection of the most plausible ICER. This method is a rank-based selection process which selects the ICER in the following order:

1. The ICER clearly adopted by NICE for decision making purposes;
2. The estimate given by NICE’s Decision Support Unit (in cases where the DSU was consulted);
3. The estimate given by the Evidence Review Group (ERG) report;
4. The estimate provided by the manufacturer.

The SMC also often presents more than one ICER in appraisals (i.e. sensitivity analyses, changes in the modelling). However, since the decision is taken based on what appraisal committee consider the most plausible ICER after a detailed discussion of the estimate provided by the manufacturer, rather than an estimate produced by an independent review group, we selected that estimate. For both NICE and the SMC, the ICERs in appraisals take into account confidential discounts offered through ‘Patient Access Schemes’.

## Results

### Novel drug approvals by designation and indication

Table 1 gives details of the NDAs made by the FDA for the period 2011–2015. It can be seen that around 40% of all approvals were for orphan drugs and that around 50% of all approvals were oncology products.

**Table 1:** Distribution of novel drug approvals by designation and indication. Source: FDA, Novel Drugs Approvals 2011–2015

	Non-oncology	Oncology	Total
Orphan drugs	36	35	71
Non-orphan drugs	58	53	111
Total	94	88	182

### Research and development cost

**Fig. 2:**
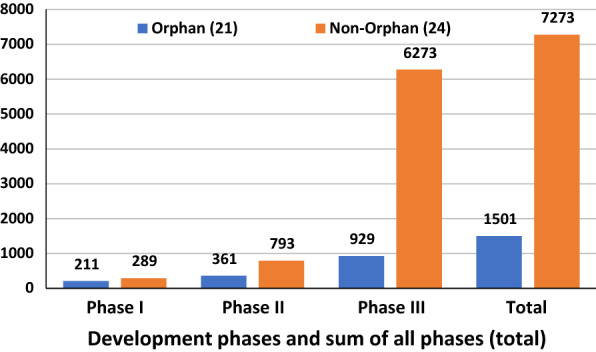
Average number of patients by orphan and non-orphan designation for all indications. Oncology drugs are included in all indications (Source: ClinicalTrials.gov)

Based on the data for 2015 novel drug approvals, a significantly greater number of patients were enrolled in clinical trials for non-orphan drugs as compared to orphan drugs. This is as expected; orphan drugs target rare diseases, so the size of the treatment populations must be lower. Therefore, the average sample size for the clinical trials is also likely to be lower, because of the challenges of recruiting patients. The main difference occurs in phase III trials, where the effectiveness of the drug is typically tested on larger samples of patients in order to demonstrate a statistically significant difference in relative treatment effect. Figure 2 shows differences both by development phase and in total.^14^

**Fig. 3:**
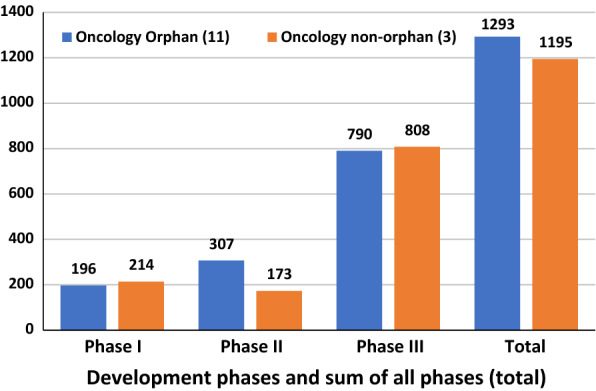
Average number of patients by orphan and non-orphan designation for oncology medicines (Source: ClinicalTrials.gov)

However, for oncology products no significant differences in patient numbers were observed between medicines designated orphan or non-orphan, suggesting that the evidentiary standards are similar across all cancer indications. See Fig. 3.

The size of clinical trials is one of the main determinants of the out-of-pocket cost of developing a drug and therefore the R&D cost of developing an orphan drug must be lower than that of a non-orphan drug.

The other factor we take into account is the difference in the overall development success rate (defined in terms of the proportion of drugs obtaining a market authorization from FDA), which is a key driver of overall R&D cost. As per our database, this was 34.6% for orphans and 5.9% for non-orphans. This difference is driven by oncology indications, where the orphans’ cumulative success rate was 28.5%, but only 2.4% for oncology non-orphan drugs. One can only speculate why this might be the case. One possibility is that targeting smaller patient populations, often based on a gene expression test, increases the chances of success.

Using our estimates of out-of-pocket costs and success rates from the literature, we estimated the R&D costs based on the model developed in [12]. These estimates, set out in Table 2, should be viewed with caution as they are based on a small sample. However, they do suggest possible differences in the R&D costs for orphan and non-orphan drugs.^15^

**Table 2:** Estimated R&D cost of a new drug FDA (US$ millions). Source: Authors’ calculations

	Orphan drugs	Non-orphan drugs	% of orphans to non-orphans
All indications	501.2	2140.8	23.4
Oncology	511.6	2341.5	21.8

Estimates have been calculated using data from 2015 novel drug approvals of FDA; Estimates for all indications include oncology products

One point to note when considering the results in Table 2 is that the R&D cost of a non-orphan drug for oncology indications is near five times the R&D cost of an orphan drug for oncology, despite the number of patients in clinical trials being similar (Fig. 3).^16^ This reflects the lower probability of success, so more non-orphan oncology projects need to be started to achieve one successful licensed product.

Overall, we estimated the R&D cost of an orphan to be around the 23% of the cost of a non-orphan, which is in line with what is argued in [9], and close to what is reported in [25, 26] whose estimates of the phase III cost of an orphan are around a 25% of the phase III cost of a non-orphan. In order to estimate the relative lifecycle costs of producing orphan and non-orphan drugs it is necessary to determine whether all the other costs (in manufacturing, marketing and distribution) are reduced by a corresponding amount. If that is not the case, it would be necessary to estimate what proportion R&D costs are of the total.

The newest estimate we have found in the literature shows that R&D costs represent 34% of total lifecycle costs [27] which is in line with the 30% figure in [28], a study published in 1997. Therefore, for the base case estimate we produced adjustments of cost-effectiveness thresholds by applying Eqs. (5) and (6) based on two alternative assumptions: (i) for ultra-orphans we assume that all drug lifecycle costs were reduced by the same proportion as R&D costs, and (ii) for regular orphans only 34% of lifecycle costs were reduced by 23.3% (or 21.8% for oncology drugs), the remainder being equivalent for regular orphan and non-orphan drugs. The first approach produces more conservative estimates, as the greater adjustment to the drug lifecycle cost cancels out a greater proportion of the revenue adjustment in our formula, thereby resulting in a lower adjusted CET. However, because we are not sure which assumption is more appropriate, we present the adjusted CETs for orphan and ultra-orphan drugs resulting from both approaches in a sensitivity analysis.

Resulting average patient population per 50,000 inhabitants for an orphan drug, 2.54 in SMC appraisals and 2.91 in NICE appraisals, is quite similar. However, the same figure for non-orphans, 82.8 in SMC appraisals and 102.57 in NICE appraisals, is 25% higher in England than in Scotland. This may be due to the small sample size, the different subsets of drugs appraised by the two organisations, or other country-specific demographic or epidemiologic factors. However, for both SMC and NICE, the average patient population is much lower for orphan drugs. Assuming that potential revenues are related to patient populations, a reasonable price for orphans would need to take account of these differences.

### Estimating the reasonable price for an orphan drug

Using our estimates of differences in R&D costs and treatment populations for orphan and non-orphans and applying our proposal of a reasonable price as set out in Eqs. (5) and (6), we can estimate adjusted CETs corresponding to orphan and ultra-orphan drugs. Although estimates of orphan and non-orphan population sizes presented in Table 3 show some degree of variability between SMC and NICE, for the base case we use the NICE estimate of non-orphan patient population size for the adjustments, rounded to 100 per 50,000 inhabitants. In a sensitivity analysis we explore the impact of different assumptions about the size of the non-orphan patient population.

**Table 3:** Non-orphan, orphan and ultra-orphan population sizes. Sources: EMA, NICE and authors calculations

	Standardised per 50,000	Absolute
Orphan cut-off population	25	26,932
Orphan mid-point population	12.5	13,462
Ultra-orphan cut-off population	1	1077
Non-orphan average	100	107,732

Adjustments of the CET have been made for both orphan designation and ‘ultra-orphan’ drugs. For the adjustment of the revenue for orphans and ultra-orphans, we have calculated the average adjusted CETs taking the ‘cut-off’ point populations of orphan and ultra-orphan drugs as well as the mid-point cut-off orphan population^17^ and average non-orphan population used in NICE appraisals. These population sizes are set out in Table 3, standardised by population and in absolute numbers. To estimate the absolute populations, we first multiply standardised numbers by the UK population (less Scotland) in NICE technology appraisals and, second, we divide the resulting number by 50,000.

To estimate the adjusted CET applying (5) and (6) we have made several assumptions which enable us to estimate the adjustment for the UK:

1. The £20,000/QALY threshold currently used by NICE is the appropriate starting point.
2. The corresponding weights of global R&D cost and operational variable cost relevant to the UK market are proportional to the UK’s market share of global pharmaceutical sales.^18^
3. Patent expiration time is 10 years after market launch time ($$T_{Ex} = T_{L} + 10)$$.
4. Non-orphan drugs only achieve 50% of their potential sales due to in-class competition.^19^
5. A discount rate of 11% is used for revenues^20^ (same as used for the estimation of R&D cost).

R&D costs, other costs and revenues (populations) have been discounted to the present value at the time period ($t = 0$) when research starts.

**Fig. 4:**
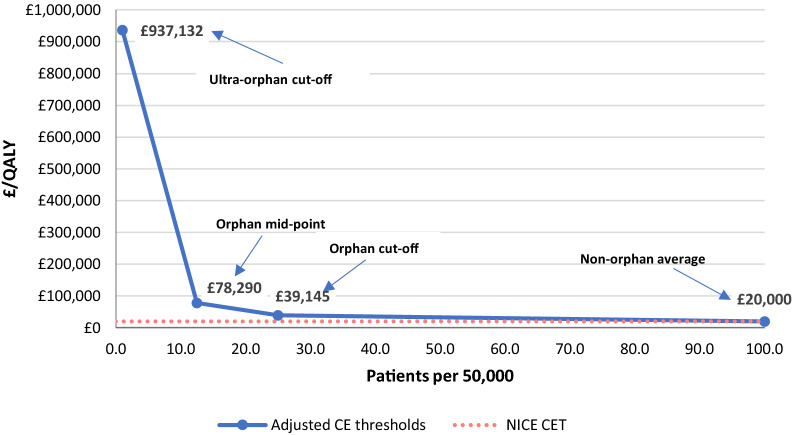
Adjusted cost effectiveness thresholds (Source: Authors calculations)

Figure 4 shows the adjusted the CETs for three different population sizes of orphan drugs shown in Table 3: orphan cut-off population, orphan mid-point population and ultra-orphan cut-off population. Using the NICE incremental cost-effectiveness threshold (£20 K per QALY) as an anchor and adjusting by R&D costs and expected market revenue, we estimated the adjusted reasonable CET for orphan drugs to be £39.1 K per QALY at the orphan population cut-off and £78.3 K per QALY at the orphan population mid-point. For ultra-orphan drugs the adjusted CET resulted in £937.1 K. To calculate the ultra-orphan cut-off point adjusted CET, we make the conservative (lower CET) assumption that the operational costs (e.g. manufacturing, marketing, commercialisation) decrease in the same proportion as the R&D cost decrease.

We also have calculated adjusted CETs for oncology products for the same three population sizes but adjusting by their specific R&D cost as shown in Table 2. Adjusted CETs for oncology orphans were almost equal to the adjusted CETs for all orphans: £41.4 k for orphan cut-off population, £82.9 k for orphan mid-point population and £1107.4 k for ultra-orphan cut-off population. The similarity of results is because the weight of the R&D cost in determining the reasonable price is relatively low compared to the weight of population size.

### Actual decisions made by NICE and SMC

Table 4 shows the average ICERs of the orphan and non-orphan drugs appraised by both NICE and SMC, for all the drugs appraised and for those that were recommended.^21^

**Table 4:** Average ICER of orphan and non-orphan drugs. Sources: SMC https://www.scottishmedicines.org.uk/; NICE https://www.nice.org.uk/

	SMC	NICE
Orphan drugs (all)	£68,064	£73,530
Non-orphan drugs (all)	£24,090	£24,840
Orphan drugs (recommended)	£45,622	£43,918
Non-orphan drugs (recommended)	£22,813	£24,207

In general, the ICERs for orphan drugs are higher than the ICERs for non-orphans. Considering only the recommended drugs, the same pattern emerges, but with a smaller difference in the ICERs for orphans and non-orphans. For the recommended drugs the ICERs of orphans are almost 2 times higher for orphans than for non-orphans.

These data suggest that both organizations may be implicitly adjusting their willingness to pay for medicines that target rare diseases, although in the case of NICE the decisions made on oncology drugs (orphan and non-orphan) will also be influenced by application of the End-of-Life (EoL) guidance.

**Fig. 5:**
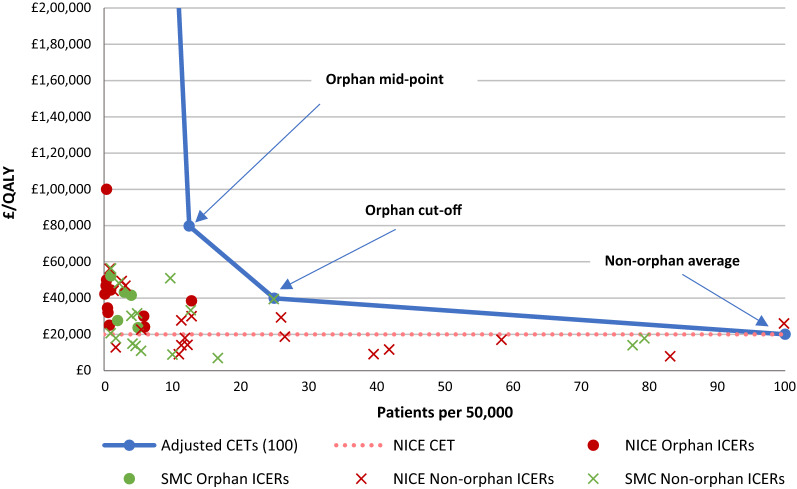
Adjusted cost-effectiveness thresholds and NICE-SMC decisions to recommend. The line that relates population sizes to adjusted CET by connecting our adjusted CETs of this figure goes to the point of the ultra-orphan CET (£937 k/QALY) (Sources: NICE (https://www.nice.org.uk/), SMC (https://www.scottishmedicines.org.uk/) and authors’ calculations)

In Fig. 5 we have superimposed our adjusted cost-effectiveness thresholds on the actual decisions by the SMC and by NICE in order to see how the proposed adjusted thresholds from our approach compare with SMC’s and NICE’s decisions. All observations for non-orphans whose populations exceed the orphan population cut-off point, present ICERs below (or quite close to) the standard cost effectiveness threshold.^22^

To assess the impact of the assumptions made to estimate the adjusted CETs, a sensitivity analysis was conducted. Additional to the impact of the assumptions made on the different population sizes of non-orphans, orphans and ultra-orphans, we also explored the impact on the results of other assumptions applied, in particular, those regarding the market exclusivity period for orphan drugs and the degree of in-class competition. None of the assumptions have a major impact on results. The assumption about the degree of in-class competition for non-orphans, implying a reduction of the population size of 50%, has the largest impact. Details of the sensitivity analysis are provided in Appendix 4.

## Discussion

Our analysis does not indicate what society *should* be prepared to pay for an orphan drug. Rather, in this paper we propose a method for establishing a reasonable price for an orphan drug in situations where a value-based price is deemed inappropriate. The method rests on the proposition that, although societal decision-makers may be willing to pay above their standard value-based price to make treatments for some orphan diseases available, they would still need a benchmark for use in price negotiations. One possible guide for setting prices would be to ensure that the manufacturers of orphan drugs do not make higher profits than manufacturers of drugs for non-orphan conditions. In order to establish a price based on this proposition, we have examined, for illustrative purposes, how the standard incremental cost-per-QALY cost-effectiveness threshold (CET) in the UK would need to be adjusted to reflect typical differences between orphan and non-orphan products in both (i) the costs of R&D and (ii) in the size of the expected treatment population. Whilst we recognize that there may be concerns about how well the QALY captures the value of the health gain for some rare conditions, we regard the (cost-per-QALY) CET as an effective tool for illustrating our reasonable price approach.

It is important to stress that our analysis does not indicate what society *should* be prepared to pay for an orphan drug, since this involves important societal judgments about whether some population health in total should be forgone in order to provide funding for treatments for rare conditions and, if so, how much. Rather, our approach could be viewed as one way of determining the *maximum allowable price* society should be willing to pay, based on allowing a reasonable rate of return. Thus, it might be used as a starting point for negotiations. Awarding a lower price would send a signal to manufacturers about the level of priority being assigned to the treatment of orphan conditions. We return to this point later.

While we believe our proposed method has some merit, it is evident that further research is required in order to improve the estimates produced. First, in estimating the costs of R&D, we only sampled novel drugs approved by the FDA in 2015. A larger sample, covering more years, might have generated different estimates of the relative research costs for orphans and non-orphans. Second, post-approval R&D costs (phase IV) should be included as they may differ as between ultra-orphan, orphan and non-orphan and would imply differences in the relative R&D cost estimates. However, our estimates, showing a lower R&D cost for orphan products, are consistent with those in earlier studies [9]. In addition, we show that the difference in the research cost between orphans and non-orphans is smaller for oncology products. This is consistent with our expectations, given that the research requirements for all oncology drugs are similar, with both orphan and non-orphan products being eligible for the FDA’s ‘fast track’ programmes for innovative drugs i.e. ‘Accelerated Approval’ and ‘Breakthrough Therapy’ [29]. These programmes often grant market approval based on less mature clinical data.

Second, we made assumptions about the differences in drug lifecycle costs other than R&D. Our simplest assumption was that only R&D costs, representing 34% of the total drug lifecycle costs, would be lower, with all other costs being the same for both orphan and non-orphan drugs. An alternative approach is to assume that the costs of manufacturing, marketing or distribution differed in the same proportion as R&D costs as orphans and non-orphans. This is the most conservative approach and produces slightly lower estimates of the adjusted CETs. Marketing and distributing an orphan drug to a small group of identified individuals, may be lower per patient than for a non-orphan drug. The truth is probably somewhere between these two extremes. However, since the assumption concerning the level of reduction (if any) in non-R&D costs makes a substantial difference to the adjusted CETs, these estimates should be verified by further research.

Third, we haven’t included the tax credit of the 50% of the phase III clinical testing costs for orphan drugs included in the US Orphan Drug Act of 1983^23^ and which remained unchanged until 2018, when reduced to 27.5%.^24^ Although in principle it could have an impact on our estimates of R&D cost for an orphan drug and consequently could reduce the adjusted CET for an orphan, the numerical impact is minor, since we are apportioning global R&D costs to the UK by the global market share of pharmaceutical sales. Furthermore, the tax credit will not be applicable for drug development based in Europe and Asia. Other push incentives given by governments, governmental agencies, product development partnerships and philanthropic donors are neither included in the estimation of the R&D cost of orphan medicines nor of non-orphans. These other push incentives are not orphan drug specific and therefore we assume they have a neutral effect on the relative R&D costs of orphans and non-orphans.

Fourth, the estimates of the costs of R&D are highly sensitive to the success rates in the development of new products. In our sample of oncology drugs, the numbers of patients in the different phases of clinical research were similar for orphans and non-orphans. However, applying different estimates of the success rate (2.4% for non-orphans and 28.5% for orphans) led to differences in overall R&D cost.^25^ While there might be reasons to expect a higher success rate for orphans, given the more precise targeting of therapy, this issue requires further investigation.

Fifth, we used estimates of target patient populations given in technology appraisals performed by the SMC and NICE in the UK. These may not reflect typical patient populations for the drugs studied for two reasons. First, in the case of orphan drugs, it is possible that some would also be indicated for larger, non-orphan populations, negating the argument for an adjusted threshold to compensate for a smaller treatment population [9]. In the case of the non-orphan drugs studied, the appraisal conducted by NICE or the SMC may have focused on a sub-set of the total population for the licensed indication for the drug concerned. For example, for oncology products in particular, it is common for a technology appraisal to focus on a given stage of disease, even if the product is licensed for other stages.

This issue was harder to investigate, but we did note that a small number (10) of the non-orphan oncology drugs in our sample had estimated patient populations in the appraisals that were lower than those for many orphan drugs in the sample.^26^ Therefore, if patient population sizes were to be used as part of an argument to allow an adjusted threshold for orphan drugs, such a policy would require increased accuracy in the estimation of target patient populations and an understanding that the eligibility of an orphan drug for an adjusted threshold could be lost if the total patient population size were to increase beyond that typically designated ‘orphan’. Additionally, the policy should be designed to prevent potential perverse incentives to strategically narrow/stratify the scope of patients licenced in order to obtain higher prices. One approach to tackle any perverse incentives to expand patient populations by either approving medicines for multiple orphan and non-orphan indications and/or strategically narrowing/stratifying patient populations, would be to take the cumulative total patient population across all indications for the CET adjustment.^27^

Sixth, we are aware that all drug prices, orphan and non-orphan, are subject to confidential discounts in many settings. Since the method described here establishes a reasonable price through an adjustment of the CET, the price that the policy maker of each country actually pays would be the one used to consider whether the drug was cost-effective, and not the published list price. However, we cannot observe confidential discounts. The existence of confidential discounts would only be problematic for our analysis if they were different as between the two types of drug. We have no reason to expect them to differ, so we make the assumption that they do not.

Seventh, we have used patient population sizes as a predictor of the likely revenue generated from the sales of the various products in the sample, at the price implied by the adjusted threshold in each case. However, it could be the case that the market exclusivity granted to orphan products means that revenue generation could be maintained for a given patient population for a longer period than that non-orphan drugs, since the latter would be more vulnerable to the entry of new, competitor products, including generics. Therefore, in the base case we assumed that a non-orphan product would only be used in 50% of the total patient population, due to the emergence of competitors.

The use of so many assumptions means that there is considerable uncertainty around the estimates of the adjusted CETs our method has produced. This uncertainty is explored in the sensitivity analysis. However, if our method were applied in practice, it may be feasible to obtain more accurate data for many of the parameters. We propose that industry wide data on R&D costs be used, together with bands of population size. Adjusting CETs by bands of patient population sizes avoids a ‘cost-plus’ pricing policy based on a company’s actual costs, which would give no incentives for efficiencies in research and development, be hard to verify, and need, somehow, to take account of failures.

Also, although we have suggested potential adjustments to the threshold ICER for orphan drugs, we have retained the standard cost per QALY rubric. Despite orphan status, it seems reasonable to expect products to show evidence of QALY gains, thereby maintaining the principle of assessing cost-effectiveness. However, some would argue that it is unreasonable to require orphan drugs to meet the same evidential standards as non-orphans, given their smaller patient populations and the likely lower level of understanding of the disease process [30]. This may be the case for many ultra-orphan products, but our research shows that oncology orphans often have trial population sizes equivalent to non-orphan cancer drugs. Furthermore, the trend for the FDA and EMA to offer various ‘accelerated approval’ programmes means that many oncology products, both orphan and non-orphan, will be licensed based on less extensive clinical data.

Finally, adopting this approach for establishing a reasonable price for an orphan drug does not tackle the broader issue of determining appropriate research priorities for the development of orphan drugs, or drugs in general. A value-based pricing policy not only ensures that the therapies adopted by the health care system are good value for money; it also encourages a shift in manufacturer research strategy towards delivering products that produce high added value in population health terms. Determining a reasonable price for the orphan drugs based on allowing a reasonable rate of return does not, of itself, appropriately drive the direction of future research. One could argue that allowing a higher CET for specialized services, as in the English NHS, but to limit this to £300,000 per QALY, is one way of sending such a signal to manufacturers. Namely, society may be willing to offer a reward to manufacturers for developing drugs for rare conditions, but this reward may not be as high as that to manufacturers developing drugs that have a major impact on population health.

This is a societal value judgment that we are not qualified to make. However, according to our data, the currently proposed CET for ultra-orphans in the UK—at its maximum of £300 k per QALY—would guarantee the average industry rate of return for most orphan drugs with patient populations as low as 3.2 per 50,000, but not for the target populations for drugs designated ‘ultra-orphan’ (i.e. 1 per 50,000 individuals). Therefore, there remains a case for having more discussion of priorities for research into rare diseases, given the large number of very rare diseases for which drugs could potentially be developed.

## Conclusions

Our research proposes one method for establishing the reasonable price for an orphan drug, based on the proposition that the expected return for developing an orphan should be no greater than the industry average. Assuming prices for drugs are set according to value added, the method proposes the adjustment that would need to be made to a payer’s “normal” cost-effectiveness threshold (CET) for non-orphan drugs in order to ensure that orphan drug developers achieve the industry-wide rate of return.

Our estimates of adjusted CETs—by the R&D cost and expected revenues—establish that, using our data sources and assumptions, the cost-effectiveness threshold for orphans would need to be higher in order to secure a price for orphan drugs that enables the manufacturer to achieve a rate of return equivalent to that from non-orphan drugs. Furthermore, the threshold would also need to increase as the targeted patient population size decreases.

Further research is required, to improve the estimates and assumptions of key parameters (i.e. other relative operational costs, treatment populations sizes, average health gains, relative direct cost savings, degree of in-class competition for orphan and non-orphan drugs, etc.). In addition, society still needs to tackle the broader issue of determining appropriate research priorities for the development of orphan drugs.

Finally, results do not indicate what society *should* be prepared to pay for an orphan drug, since this involves societal judgments about whether some population health in total should be forgone in order to provide funding for treatments for rare conditions. Our research should be viewed as one way of determining the *maximum* price society should be willing to pay to ensure a reasonable rate of return.

## Figures and Tables

**Fig. 6:**
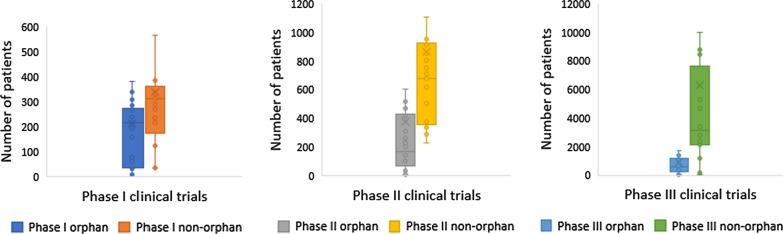
Number of patients in clinical trials per development phase. Outliers have been removed form the box-and-whisker plots (Source: Clinicaltrials.gov)

**Fig. 7:**
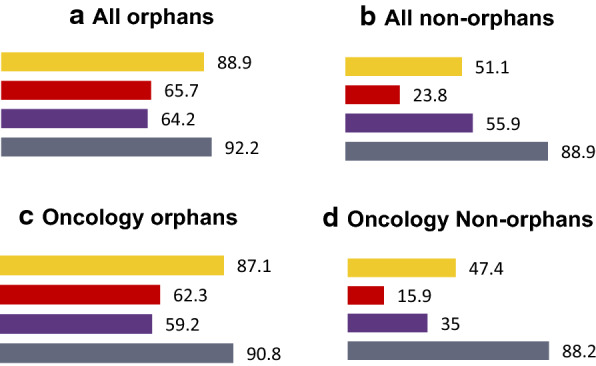
Probabilities of Success in percentage (%).  Phase 1;  phase 2;  phase 3;  NDA/BLA review and approval (Source: Biomedtracker | Informa, 2019)

**Fig. 8:**
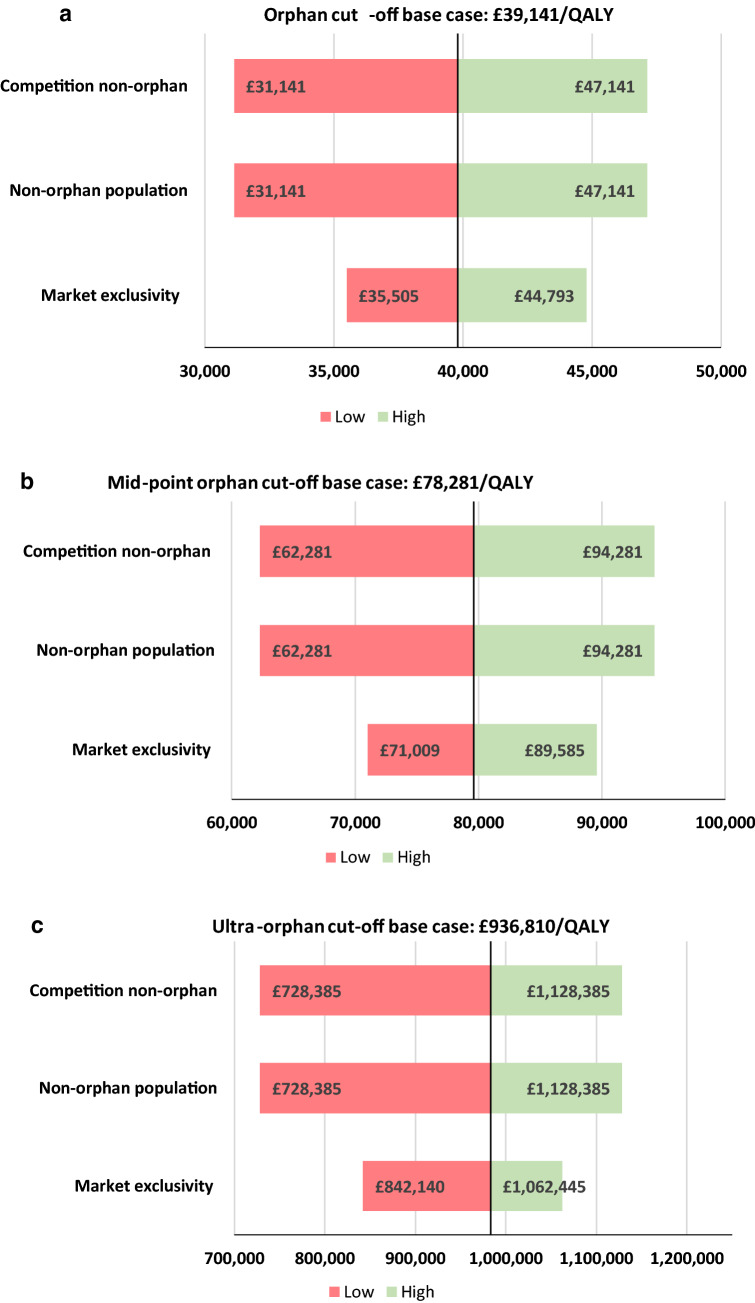
Sensitivity analyses: variations in absolute values. **a** Variance on the adjusted CET for orphan cut-off; **b** variance on the adjusted CET for mid-point of the orphan cut-off; **c** variance on the adjusted CET for ultra-orphan cut-off (Source: Authors calculations)

## Data Availability

Five appendices have been included after references section. The first four are key to show and interpret the results, as well as for supporting the “Discussion” and “Conclusion” sections of the paper. Authors’ preference would be two include Appendix 1, which contains the formal development of our approach for a reasonable price for an orphan drug, either in the main body of the paper or as an appendix section (as it is now). We think that showing the algebra supporting our approach is important to understand, in an ideal situation what we are proposing in this research as a reasonable price. Appendix 2 shows variability in the number of patients in trials using box-and-whisker plots. Data in plots are for all indications only. Appendix 3 shows graphically and in colour code the probability of success data we have retrieved from the Biomedtracker, Pharmapremia database for the period 9/2009–9/2019. For Appendix 4, contains the sensitivity analyses. Results of different sensitivity analyses performed are shown in tables and figures. For appendix 5 shows a table with the 10 non-orphan oncology products which have patient populations lower than those for many orphan drugs in the sample. Regarding the data used for the study, all have been obtained from publicly available datasets from the following sources: Information of Novel Drug Approvals 2011–2015 has been obtained from FDA webpage at https://www.fda.gov/Drugs/DevelopmentApprovalProcess/DrugInnovation/ucm592464.htm Information of number of patients has been obtained from ClinincalTrials.gov at: https://clinicaltrials.gov/ Health technology appraisals containing data of intended to treat patient populations, ICERs and recommendation status have been obtained from NICE and SMC websites at https://www.nice.org.uk/ and https://www.scottishmedicines.org.uk/ respectively. Data of probabilities of success have been obtained from the Biomedtracker, Pharmapremia database for the period 9/2009–9/2019. We have created our dataset for all analyses performed for this research by combining information coming from the three sources cited above, as well as from the literature appropriately cited along the main text. The dataset used and/or analysed during the current study are available from the corresponding author on reasonable request.

## Appendix 1

For a formal development of our approach consider the following notation:

- $$t = 0$$: research starts
- $P_{D}$: patent or other form of exclusivity duration in number of years
- $t = T_{P}$: point of patenting
- $t = T_{L}$: point of market launch
- $t = T_{Ex} = T_{P} + P_{D}$: point of patent expiration or other form of exclusivity
- $t = 0, \ldots ,T_{P,} \ldots ,T_{L} , \ldots , T_{Ex}$: drug’s life-cycle
- $$\delta$$: discount factor
- $P_{no}$: price non-orphan
- $$P_{o}$$: price orphan
- $P_{no/c}$: price non-orphan comparator
- $P_{o/c}$: price orphan comparator
- $$P_{no} = P_{no/c} = P_{o} = P_{o/c} = 0, \forall t \in \left[ 0 \right.,\left. {T_{L} } \right)$$
- $q_{no}^{t}$: quantity non-orphan (equal to comparator) in period t
- $q_{o}^{t}$: quantity orphan (equal to comparator) in period t
- $$q_{no}^{t} = q_{o}^{t} = 0, \forall t \in \left[ 0 \right.,\left. {T_{L} } \right)$$
- $R_{no}^{t}$: R&D cost of a non-orphan in period t
- $R_{o}^{t}$: R&D cost of an orphan in period t
- $$R_{no}^{t} = R_{o}^{t} = 0, \forall t \in \left( {T_{L} } \right.,\left. {T_{E} } \right]$$
- $C_{no}^{t}$: other components of the cost of a non-orphan (marketing, manufacturing, distribution) in period t
- $C_{o}^{t}$: other components of the cost of an orphan (marketing, manufacturing, distribution) in period t
- $$C_{no}^{t} = C_{o}^{t} = 0, \forall t \in \left[ 0 \right.,\left. {T_{L} } \right)$$
- $\pi_{no}$: return for a non-orphan developer (current value at t = 0)
- $\pi_{o}$: return for an orphan developer (current value at t = 0)
- $B_{no} :$ per patient health gain non-orphan
- $B_{o} :$ per patient health gain orphan
- $B_{no/c} :$ per patient health gain alternative (therapy) to non-orphan
- $B_{o/c} :$ per patient health gain alternative (therapy) to orphan
- $CET:$ cost effectiveness threshold

Let the Incremental Cost Effectiveness Ratio (ICER) for a non-orphan be:

$$ICER = \frac{{\left( {P_{no} - P_{no/c} } \right)}}{{\left( {B_{no} - B_{no/c} } \right)}}$$

Payers and/or HTA agencies fix the CET which ICER must not overcome for a reimbursement recommendation. Substituting the ICER by the CET, then we have that,

$$CET = \frac{{\left( {P_{no} - P_{no/c} } \right)}}{{\left( {B_{no} - B_{no/c} } \right)}}$$

Assuming value-based prices, a non-orphan drug developer will set the price as follows:

1 $$P_{no} = CET\left( {B_{no} - B_{no/c} } \right) + P_{no/c} .$$

Analogously an orphan developer will set the price as follows:

2 $$P_{o} = CET\left( {B_{o} - B_{o/c} } \right) + P_{o/c} .$$

Let the return for a non-orphan drug developer be:

$$\pi_{no} = \mathop \sum \limits_{t = 0}^{{t = T_{Ex} }} \delta^{t} \left( {P_{no} q_{no}^{t} - C_{no}^{t} - R_{no}^{t} } \right).$$

Analogously the return for an orphan drug developer will be:

$$\pi_{o} = \mathop \sum \limits_{t = - 0}^{{t = T_{Ex} }} \delta^{t} \left( {P_{o} q_{o}^{t} - C_{o}^{t} - R_{o}^{t} } \right).$$

Following our approach for a reasonable price of an orphan drug, both returns should be equal and therefore we have that,

3 $$\mathop \sum \limits_{t = 0}^{{t = T_{Ex} }} \delta^{t} \left( {P_{o} q_{o}^{t} - C_{o}^{t} - R_{o}^{t} } \right) = \mathop \sum \limits_{t = 0}^{{t = T_{Ex} }} \delta^{t} \left( {P_{no} q_{no}^{t} - C_{no}^{t} - R_{no}^{t} } \right).$$

Using (1) and (2) into (3) and assuming that the cost effectiveness threshold for an orphan $CET_{o}$ must be different from the $CET$ as our reasonable pricing approach establishes, we have that,

$$\mathop \sum \limits_{t = 0}^{{t = T_{Ex} }} \delta^{t} \left( {\left( {CET_{o} \left( {B_{o} - B_{o/c} } \right) + P_{o/c} } \right)q_{o}^{t} - C_{o}^{t} - R_{o}^{t} } \right) = \mathop \sum \limits_{t = 0}^{{t = T_{Ex} }} \delta^{t} \left( {\left( {CET\left( {B_{no} - B_{no/c} } \right) + P_{no/c} } \right)q_{no}^{t} - C_{no}^{t} - R_{no}^{t} } \right).$$

Rearranging we have that,

4 $$CET_{o} = \frac{{\mathop \sum \nolimits_{{t = T_{P} }}^{{t = T_{Ex} }} \delta^{t} \left( {CET\left( {B_{no} - B_{no/c} } \right)q_{no}^{t} + \left( {P_{o/c} q_{o}^{t} - P_{no/c} q_{no}^{t} } \right) - \left( {C_{no}^{t} - C_{o}^{t} } \right) - \left( {R_{no}^{t} - R_{o}^{t} } \right)} \right)}}{{\mathop \sum \nolimits_{{t = T_{P} }}^{{t = T_{Ex} }} \delta^{t} \left( {CET\left( {B_{o} - B_{o/c} } \right)q_{o}^{t} } \right)}}.$$

where $CET_{O}$ represents the adjusted cost effectiveness threshold for an orphan drug. Under perfect information about health benefits, prices and quantities of new orphan and non-orphan drugs and their comparators, as well as for R&D costs and other components of the cost for orphans and non-orphans, CET could be adjusted by applying (4) to calculate a reasonable value-based price for each new orphan drug. However, collecting accurate data for all variables involved in (4) is beyond the scope of this work.^28^ Therefore, in order to apply our proposed approach to the data we have been able to collect (i.e. population sizes and R&D costs), it is necessary to make assumptions about several components of (4). We set out our assumptions below:

- Cost and health benefits for both comparators, orphan and non-orphan drugs, are equal to zero: $P_{o/c} = P_{no/c} = 0$ and $B_{o/c} = B_{no/c} = 0$.
- Equal variable costs (manufacturing, distribution, etc.) of orphans and non-orphans: $C_{no}^{t} = C_{o}^{t}$.
- Average health gain per patient for non-orphan drugs equal to one: $B_{no} = 1$.
- Equal average health gain per patient for orphans and non-orphans: $B_{o} = B_{no}$.

Applying the above list of assumptions, we can simplify (4) into:

5 $$CET_{o} = \frac{{\mathop \sum \nolimits_{0}^{{T_{EX} }} \delta^{t} \left( {CETq_{no}^{t} - \left( {R_{no}^{t} - R_{o}^{t} } \right)} \right)}}{{\mathop \sum \nolimits_{0}^{{T_{EX} }} \delta^{t} q_{o}^{t} }}$$

where $CET$ is the Cost Effectiveness Threshold; $q_{no}$ is the average population of non-orphan, $q_{o}$ is the cut-off (alternatively the average) population of orphan; $R_{no}$ is the R&D cost of a non-orphan; and $R_{o}$ is the R&D cost of an orphan.

Additionally, for the adjustment of the CET we also want to differentiate between orphan—from now on noted by the subscript *o*—and ultra-orphan drugs—from now on noted with the subscript *uo*. For this purpose, we want to make a specific assumption for ultra-orphans:

- Variable costs (manufacturing, distribution, etc.) of ultra-orphans are lower than variable costs of non-orphans, in the same proportion as the R&D costs: $C_{no}^{t} > C_{uo}^{t}$

Applying this additional specific assumption for ultra-orphans to (4) and keeping all other assumptions for orphan drugs constant we have that:

6 $$CET_{uo} = \frac{{\mathop \sum \nolimits_{0}^{{T_{EX} }} \delta^{t} \left( {CETq_{no}^{t} - \left( {C_{no}^{t} - C_{uo}^{t} } \right) - \left( {R_{no}^{t} - R_{uo}^{t} } \right)} \right)}}{{\mathop \sum \nolimits_{0}^{{T_{EX} }} \delta^{t} q_{uo}^{t} }}$$

where, in addition to (5), $q_{uo}^{t}$ is the cut-off (alternatively the average) population of ultra-orphan in period t; $C_{no}$ is the variable cost of a non-orphan; $C_{uo}^{t}$ is the variable cost of an ultra-orphan in period t; and $R_{uo}^{t}$ is the R&D cost of an ultra-orphan in period t.

## Appendix 2

See Fig. 6.

## Appendix 3

See Fig. 7.

## Appendix 4

The impact of the several assumptions made for the adjustment of the CETs was assessed by performing different sensitivity analyses. One feature of orphan drugs is the absence of competitors during the patent period. This is due firstly because of rarity, which hinders the development of treatments and therefore the likelihood of other competitors for the same disease,^29^ and secondly because of the market exclusivity period new orphan drugs are granted in the US (7 years)^30^ and the EU (10 years).^31^ This is a different context than the one characterising non-orphan drugs developed to treat common diseases, where competition is common place and drugs have to compete for market share with other in-class competitors.

For the estimation of the base case CETs we assumed a length of 10 years of market exclusivity for orphan drugs and a 50% of market share for non-orphans. Additionally, for the estimation of the CETs we assumed the populations in Table 5 of this paper. Therefore, we conducted a sensitivity analysis of the impact of these assumptions on the adjusted CET, using the ranges shown in Table 6.

**Table 5:** Assumptions and variations for sensitivities

Sensitivity	− 20%	Base case	+ 20%
Non-orphan population size^a^	80	100	120
Competition in non-orphan markets^b^	40%	50%	60%
Market exclusivity of orphans	8 years	10 years	12 years

^a^Values standardised per 50,000 inhabitants

^b^Values of market share

The sensitiveness of CETs to the six assumptions assessed is presented in Fig. 8 in absolute terms.

As Fig. 8 shows, the impact of assuming different lengths for the market exclusivity period for orphans is the lowest. This is because the way in which our algebraic approach works minimises the impact of the market exclusivity assumption.^32^ The sensitivity of the CETs to the rest of the assumptions seems to be proportional, and constant across for each CETs (orphan cut-off, mid-point of the orphan cut-off, ultra-orphan cut-off). Changes of equal measure and sign around the assumptions of non-orphan average populations and in-class competition for non-orphans also produce equivalent variations as Fig. 8 shows. This is because both have the same impact on the final aggregate non-orphan treatment populations.

Additionally, we assess the sensitivity of the adjusted CET for ultra-orphan drugs to the assumption that other components of the life-cycle cost of ultra-orphans (e.g. commercialisation, manufacturing, distribution) go down in the same proportion than the R&D cost. Table 6 compares the adjusted CETs for both, orphan and ultra-orphan drugs, under the two different assumptions applied to lifecycle costs other than the R&D. Estimates in the table show that the assumption represents a minimum impact. For orphans, assuming all costs decrease in the same proportion reduces the adjusted CET by 4.4% (£1726). For ultra-orphans, assuming that only the R&D costs decrease while other life-cycle cost components remain constant increases the adjusted CET by 4.4% (£43,149).

**Table 6:** Sensitivity of the adjusted CET for ultra-orphans to the R&D and other life-cycle costs assumption. Source: authors’ calculations

	All costs decrease in the same proportion than the R&D cost	Only R&D costs decrease for ultra-orphan drugs
Orphan cut-off point	£37,534	£39,260
Ultra-orphan cut-off point	£938,358	£981,507

Base case estimates are shown in italics

## Appendix 5

See Table 7.

**Table 7:** Non-orphan oncology drugs and indications. Source: EMA and NICE

Name	Indication
Lonsurf	To treat adult patients with metastatic colorectal cancer (CRC) who have been previously treated with, or are not considered candidates for, available therapies including fluoropyrimidine-oxaliplatin- and irinotecan-based chemotherapy, an anti-VEGF biological therapy, and an anti-EGFR therapy
Kadcyla	To treat (adult) patients with HER2-positive, unresectable locally advanced or metastatic breast cancer who have received prior treatment with trastuzumab and a taxane
Xofigo	To treat men with symptomatic late-stage (metastatic) castration-resistant prostate cancer that has spread to bones but not to other organs
Inlyta	To treat patients with advanced kidney cancer (renal cell carcinoma) who have not responded to another drug for this type of cancer
Perjeta	To treat patients with HER2-positive late-stage (metastatic) breast cancer in combination with Herceptin and docetaxel
Xtandi	To treat men with late-stage (metastatic) castration-resistant prostate cancer who have received docetaxel therapy
Zytiga	In combination with prednisone (a steroid) to treat patients with late-stage (metastatic) castration-resistant prostate cancer who have received prior docetaxel (chemotherapy)

## Footnotes

1 According to the European Medicines Agency's (EMA), rare diseases are defined as life-threatening or chronically debilitating conditions that affect no more than 5 in 10,000 people in the EU (25 in 50,000 people). Orphan designations are granted by EMA to medicines that target to treat rare diseases. See: http://www.ema.europa.eu/ema/index.jsp?curl=pages/regulation/general/general_content_000029.jsp&mid=WC0b01ac0580b18a41. In addition, the Scottish Medicines Consortium (SMC) and the National Institute for Health and Care Excellence (NICE) define ultra-orphan drugs as medicines that have been granted by the EMA with the orphan status and target to treat conditions with a prevalence of 1 in 50,000 people in England and/or Scotland. See: https://www.scottishmedicines.org.uk/media/2782/pace-overview-document.pdf.

2 We focus on the number of patients as the main driver of clinical trials costs to account for potential differentials of R&D costs that we use for the adjustment. However, we acknowledge that clinical trials for orphan medicines are likely to be more difficult to run than clinical trials for non-orphan medicines. Recruiting rare disease and ultra-rare disease patients could be extremely difficult, imposing additional costs. Not considering these costs implies that the adjustment of the CET estimated in this work is conservative, given that we only consider the differences in trial patient numbers, not potential differences in the cost per patient in orphan versus non-orphan trials.

3 For a discussion of the issues involved in setting the CET see [11, 12]. Further discussion in setting CET in any individual jurisdiction in order to signal the optimal amount of R&D is given in [13].

4 There is always the possibility that the size of a country’s market is not driven by the appropriate CET, but by a desire to free-ride on paying for R&D costs. Of course, it is also possible that a country is overpaying for drugs, given the underlying population willingness to pay for health gain.

5 There is no established method to apportion the global joint R&D costs of a drug between different countries in the relevant literature. Neither the global pharmaceutical companies, nor governments have a method to attribute R&D costs to each country. We applied the country market share of the total global market value as a proxy of the relevant country share of the global R&D cost as one possible approach while we recognise that there could be alternatives i.e. using the country market share of the market value of the high-income countries.

6 We use FDA novel drugs approval data for the adjustment of the R&D cost. The clinical development of new medicines is global and not subject to particular locations. The FDA database is the most comprehensive database, easily accessible and of high quality. Database provides information about orphan designation, first in class, breakthrough therapy designation, fast track, priority review. http://www.fda.gov/Drugs/DevelopmentApprovalProcess/DrugInnovation/ucm430302.htm.

7 https://clinicaltrials.gov/.

8 See [17] for a review of the literature on the topic of estimating the R&D cost of a new medicine.

9 Moore and colleagues [19] also provide cost estimates of pivotal clinical trials by several factors including number of patients. They also show that cost increases with the number of patients. Our per patient cost estimates based on [18] are lower which makes our CET estimate lower.

10 The most recent published work, Wong and colleagues [22], uses data from Trialtrove and clinicaltrials.gov in addition to Biomedtracker. The authors apply a different methodology to estimate the PoS based on total programs and transitions. Their estimated PoS are lower than ours. This means that our estimate of the R&D cost of an orphan drug is lower than the estimate that would result from applying the PoS figures in [22].

11 We use NICE and SMC technology appraisal (TA) data to adjust the CET by treatment population size (revenue) because they often include treatment population data and budget impact analysis with a 1 to 5 years of time horizon. In addition, there is a wide overlap between FDA’s novel drug approvals and drugs whose TAs by NICE and SMC include treatment population data.

12 'Mid-2013 Population Estimates for Clinical Commissioning Groups (CCGs) and NHS area teams in England by Single Year of Age and Sex (experimental statistics) and NHS Area teams' for the total population of England: http://www.ons.gov.uk/ons/search/index.html?newquery=01currccg.

13 Mid-year 2016 estimate from National Records of Scotland (NRS): https://www.nrscotland.gov.uk/statistics-and-data/statistics/statistics-by-theme/population/population-estimates/mid-year-population-estimates/mid-2016.

14 For a depiction of variability in the number of patients in trials see Appendix 2 where box-and-whisker plots are shown in Fig. 6. Appendix 2 shows data for all indications only.

15 Estimates of the R&D cost of an orphan drug do not take account of any form of push incentives given in the US by the Orphan Drug Act of 1983 in the form of federal grants to subsidize drug R&D investments and tax credits.

16 Data of PoS for orphan and non-orphan oncology indications are available in Appendix 3.

17 Orphan mid-point population is the median point between the orphan population cut-off point and zero.

18 Data of 2015 market share of the UK has been taken from the IMS World Review Executive™ 2016.

19 We assume same length for patent for non-orphan drugs but without market exclusivity.

20 Since we are basing the adjustment of the CET on the need to guarantee orphan drugs developers the same rate of return of the industry’s average, a discount rate of 11% is used—the same used by private organisations for their financial calculations.

21 The purpose of considering ICERs from both SMC and NICE was not to make comparisons of ICERs between the two organisations, but to make comparisons between the ICERs for orphan and non-orphan drugs based on each data source. The use of data from two different sources provides a better indication of whether there is a consistent pattern between the ICERs of orphan and non-orphan drugs.

22 Outlier observations showing either too large populations (all non-orphans) or too large ICERs (all ultra-orphans) have been excluded from the graph in order to keep a comparable scale. Five outliers have been excluded.

23 See: https://www.fda.gov/forindustry/developingproductsforrarediseasesconditions/howtoapplyfororphanproductdesignation/ucm364750.htm.

24 See page 805 in the document available at: https://docs.house.gov/billsthisweek/20171218/CRPT-115HRPT-466.pdf.

25 See Appendix 3 for a detailed information about clinical success rates per phase of development.

26 A table with the 10 oncology products is shown in Appendix 5.

27 The use of such an approach should be subject to revision over time since, if implemented as a ‘one time’ policy for a particular orphan drug, it could generate perverse incentives for companies to use the profit maximising strategy of not developing the medicine for additional indications if that would threaten the medicine’s orphan designation or adversely affect its price as calculated by this method. This would negatively affect patients’ health.
